# The impact of poor dental status and removable dental prosthesis quality on body composition, masticatory performance and oral health-related quality of life: a cross-sectional study in older adults

**DOI:** 10.1186/s12903-022-02103-7

**Published:** 2022-04-27

**Authors:** Siraphob Techapiroontong, Nareudee Limpuangthip, Wacharasak Tumrasvin, Jirad Sirotamarat

**Affiliations:** 1 Faculty of Dentistry, Chulalongkorn University, 34 Henri-Dunant Rd, Patumwan, Bangkok, 10330 Thailand; 2 Department of Prosthodontics, Faculty of Dentistry, Chulalongkorn University, 34 Henri-Dunant Rd, Patumwan, Bangkok, 10330 Thailand

**Keywords:** Bone mass, Denture quality, Masticatory function, Muscle mass, Multiple sieve method, Tooth loss, Visceral fat

## Abstract

**Background:**

To determine the impact of dental status, types, and quality of dental prostheses on body composition, masticatory performance and oral health-related quality of life (OHRQoL). Potential associations between body composition, masticatory performance and OHRQoL were also investigated.

**Methods:**

This cross-sectional study included 110 older adults who received prosthodontic treatment at the Dental Faculty Clinics at Chulalongkorn University. Participants were categorized according to their dental prostheses: complete denture (CD), removable partial denture (RPD) and fixed partial denture (FPD). Retention and stability of the RPD and CD were evaluated using the CU-modified Kapur and the modified NHANES III criteria to classify denture quality into acceptable and unacceptable. Dental status including posterior occluding pairs and number of remaining natural teeth were assessed intraorally. Dependent variables were body composition, masticatory performance and OHRQoL. Body composition, including muscle mass (kg), bone mass (kg), basal metabolic rate (kcal) and visceral fat (%) were determined through a bioelectrical impedance analysis. Masticatory performance was assessed using a multiple sieve method of peanut mastication. OHRQoL was assessed using the validated Thai version of Oral Impacts on Daily Performances (Thai-OIDP) index. After adjusting for covariates, including age and sex, the associations between oral and dental prosthesis status and body composition, masticatory performance as well as OIDP score were analyzed using multivariable linear and negative binomial regression analyses. Spearman’s correlation was used to determine the potential associations between body composition, masticatory performance and OHRQoL.

**Results:**

The presence of fewer natural teeth or wearing an unacceptable removable denture were factors associated with lower bone mass, muscle mass and basal metabolic rate, and with a higher visceral fat. Similar dental and removable denture status were also associated with larger peanut particle size and higher OIDP score. Masticatory performance and OHRQoL variables were moderately correlated (Spearman's rho = 0.44). However, body composition was not correlated with masticatory performance or OHRQoL.

**Conclusions:**

In individuals wearing dental prostheses, factors such as severity of tooth loss, types, and quality of dental prostheses, particularly retention and stability, negatively impacted not only masticatory function and OHRQoL, but also their overall body composition and health.

**Supplementary Information:**

The online version contains supplementary material available at 10.1186/s12903-022-02103-7.

## Background

Dental caries and periodontal disease are the major causes of tooth loss [1], leading to declined masticatory function [2] and oral health-related quality of life (OHRQoL) [3]. When tooth loss occurs, patients tend to alter their dietary quality and reduce overall nutrition intake, which places them at high risk for cardiovascular disease and diabetes [4] or relevant comorbidities such as sarcopenia and osteopenia [5, 6].

Dental prostheses are necessary to improve masticatory function and OHRQoL [7, 8]. Significant decrease in self-reported oral-related problems such as eating difficulty and pain from denture were found after the conventional prosthodontic treatment [9]. However, several denture wearers have impaired masticatory ability and quality of life after a period of denture use. This might be due to the different types and qualities of the denture [8, 10]. Therefore, evaluating masticatory function in prosthodontic patients is important to maintain their masticatory function and provide a satisfactory OHRQoL.

Tooth loss can have a negative impact on the individual’s general health [11]. Some studies reported that tooth loss is associated with an increased body fat and body mass index (BMI) [12, 13]. Apart from BMI, body composition can be used to determine individual’s general health more comprehensively [14]. Changes in body composition beyond standard limits can lead to other life-threatening blood-related comorbidities [5, 6, 15], and worsen their quality of life [16–18]. There are reports about potential associations between tooth loss and body composition [19–21]. Certain studies also assessed the associations between body composition, masticatory performance and OHRQoL [13, 22–26]. However, only single body composition has been assessed per study. Also, it is yet unknown whether there is a relationship between wearing dental prostheses, with specific types and qualities, and body composition.

Thus, the aim of this cross-sectional study was to determine the impact of dental status, types, and quality of dental prostheses on body composition, masticatory performance and OHRQoL. Secondly, we investigated the potential associations between body composition, masticatory performance and OHRQoL.

## Methods

### Study design and participants

A cross-sectional study design was employed. Participants were adults and older patients who received prosthodontic treatment from Chulalongkorn University Faculty of Dentistry clinics in Thailand. Study protocol was approved by the Human Research Ethics Committee of the Faculty of Dentistry (protocol number: HREC-DCU 2021-005) and performed in accordance with the Declaration of Helsinki Ethical Principles for Medical Research Involving Human Subjects. Participants were recruited by stratified random sampling using two strata of denture types [complete denture (CD), removable partial denture (RPD), and fixed partial denture (FPD)] and age-sex (male ≥ 65 years, male < 65 years, female ≥ 65 years, and female < 65 years). They were asked about the presence of any underlying medical condition, which defined as a group of chronic condition that can cause an interference with daily life and activity which requires long-term medical care [27]. Exclusion criteria were patients who had neuromuscular or psychological disorders, unwilling to perform peanut mastication or allergic to peanuts, or unwilling to provide any information about their past medical history and preexisting systemic conditions. Participants signed an informed consent prior to participating in this study. Our study was written according to the Strengthening the Reporting of Observational Studies in Epidemiology (STROBE) recommendations.

Sample size was estimated using G*Power 3.1.9 software. Based on the final statistical analysis models, the F test family with a statistical test of multiple linear regression: fixed model, R^2^ deviation from zero were used. Assuming a medium effect size of 0.15 and the predictor numbers of 5, a sample size of 92 was calculated with 5% type I error and 80% power. A 20% attrition rate was assumed, which led to the final sample size estimation of 110 participants in the present study.

### Independent variables

#### Dental status and types of dental prosthesis

Dental status, including number of remaining natural teeth (NT) and posterior occluding pairs (POPs), were recorded. The NT (including an implant that did not retain removable prosthesis) ranged from 0 to 28, excluding third molar, whereas the POPs ranged from 0 to 8. According to a previously reported systematic review, subjects with < 20 NT or < 4 POP had worse OHRQoL than those with ≥ 20 NT or ≥ 4 POPs, and OHRQoL was more influenced by the number of POPs [28]. Therefore, these values were used as cut-off points to categorize dental status into ≥ 4 POPs with ≥ 20 NT, < 4 POPs with ≥ 20 NT, and < 20NT, increasing from the lowest to highest tooth loss severities.

Participants were further divided into three types of dental prostheses: CD, RPD, and FPD wearers. The CD and RPD qualities, in terms of denture retention and stability, were evaluated according to the CU-modified Kapur and the modified NHANES III criteria, respectively [10, 29]. Retention and stability were focused because ill-fitting denture is a common complaint among removable denture wearers [10]. Levels of removable denture retention and stability were scored using 4-point and 3-point Likert scales, respectively. Retention of maxillary and mandibular denture was considered as acceptable when the retention score was more than 2 and 1, respectively. Meanwhile, stability was acceptable when the stability score was 2 for each denture. Quality of RPD and CD was considered as clinically “acceptable” when retention and stability of both maxillary and mandibular denture were deemed appropriate. Next, participants were categorized into “acceptable” and “unacceptable” CD or RPD wearers. The FPD group included participants who had 26 to 28 NT, and had not been wearing any removable dental prosthesis. This group served as a control and was not classified according to denture quality. Denture retention and stability were only assessed in subjects with removable dentures.

### Dependent variables

Three dependent variables were assessed which included general health (body composition) and oral health (masticatory performance and OHRQoL) indicators.

#### Body composition

Four body composition measurements including bone mass (kg), muscle mass (kg), percent visceral fat (%), and basal metabolic rate per day (kcal) were determined. Body composition measurements were assessed by using bioelectrical impedance analysis (BIA) which measures electrical impedance or resistance per volume of biological tissue [30]. For this purpose, participants stood barefoot in an upright position on a digital body composition monitor (TANITA BC-587, Corporation of America, Inc., Arlington Heights, IL, USA) and their body composition was recorded [31].

#### Masticatory performance

A multiple sieve method of peanut mastication was used to determine masticatory performance [10, 32]. Each participant sat and masticated 3 g of roasted peanut for 20 strokes which was done in triplicate. Dry comminuted peanut particles were sieved via 12 standard test sieves and vibrated on a vibratory sieve shaker at a frequency of 70 Hz for 3 min. Peanut particles passed through test sieves were collected and calculated as follows: Cumulative weight percentage of each sieve = (1 − [cumulative mass retained on that sieve and previous sieve]/total sample mass) × 100%.

To determine the median peanut particle size for each participant, a simple linear regression was plotted between cumulative weight of each sieve and diameter of the sieve (mm). Median peanut particle size was defined as the sieve diameter which 50% of comminuted particles could pass through. After mastication, the smaller peanut particle size measurement (in mm) indicated the better masticatory performance.

#### Oral health-related quality of life

The OHRQoL was determined by a face-to-face interview using the validated Thai version of Oral Impacts on Daily Performances (Thai-OIDP) [33]. The index focuses on oral or denture conditions that affect a person’s ability to carry out eight daily activities within three performances: physical (1. eat, 2. speak/pronounce clearly, 3. clean teeth/denture/oral cavity), psychological (4. sleep/relax, 5. smile/laugh/show teeth without embarrassment, 6. maintain usual emotion), and social (7. carry out work, 8. contact people). Frequency and severity of each activity, determined by five-point Likert scale, were multiplied. A summation of all activity scores was then calculated to determine the OIDP score: the higher score indicated the more severity of the oral impact and worse OHRQoL.

### Data analysis

Data were analyzed using STATA version 13.0 at a 5% significance level. Descriptive statistics were calculated to report mean (± SD) and percent distribution of the participants according to the relating variables. Cronbach’s alpha was used to determine an internal consistency of the eight OIDP items. Univariate analyses were conducted to determine the differences in body composition between patient’s characteristics, dental status, types and qualities of dental prosthesis, using independent t-test and one-way ANOVA followed by Tukey post-hoc comparison test. After adjusting for potential covariates, the association of dental status as well as types and qualities of dental prosthesis, with body composition and peanut particle size measurements were analyzed using multiple linear regression analyses. Meanwhile, their associations between independent variables and OIDP score were determined using negative binomial regression analyses. Because there was a collinearity between dental status and type of dental prosthesis, all regression analyses were separated into two models using a dental status and types with qualities of dental prosthesis as an independent variable, respectively. Spearman’s correlation was used to investigate the potential relationships between body composition, peanut particle size and OIDP score.

## Results

Participants were on average 65.0 (± 9.8) years old, and female: male ratio was 3:2. Their body composition, including bone mass (kg), muscle mass (kg), basal metabolic rate/day (kcal), and visceral fat (%) are displayed in Table 1 according to biological characteristics, oral and denture status. Cronbach’s alpha of the OIDP items was 0.76, which indicates the acceptable internal consistency of the measure [34]. Univariate analyses showed that female significantly possessed lower bone mass, muscle mass, basal metabolic rate, and visceral fat compared with male individuals. Basal metabolic rate of older adults was lower than that of younger ones. Therefore, sex and age were potential confounders for these later variables.

**Table 1:** Baseline characteristics, dental status, and type and quality of dental prosthesis across body composition measurements of the samples (N = 110)

	Frequency	Bone (kg)	Muscle mass (kg)	Basal metabolic rate (kcal)	Visceral fat (%)
	%	Mean (± s.d.)			
Overall		2.29 (± 0.45)	40.2 (± 8.7)	1200 (± 236)	9.84 (± 4.47)
Age (years)					
< 65	42.7	2.43 (± 0.45)	41.9 (± 9.3)	1270 (± 255)	9.30 (± 4.18)
≥ 65	57.3	2.19 (± 0.43)	38.8 (± 8.0)	1148 (± 209)*	10.25 (± 4.66)
Sex					
Male	40.0	2.69 (± 0.33)	49.0 (± 6.5)	1405 (± 215)	13.77 (± 3.83)
Female	60.0	2.03 (± 0.31)*	34.3 (± 3.3)*	1063 (± 126)*	7.23 (± 2.52)*
Underlying medical condition					
Absence	25.0	2.29 (± 0.44)	40.4 (± 8.5)	1196 (± 228)	9.04 (± 4.22)
Presence	75.0	2.30 (± 0.45)	40.1 (± 8.7)	1203 (± 239)	10.10 (± 4.54)
Remaining NT and POP					
≥ 20 NT, ≥ 4 POP	39.1	2.37 (± 0.46)	41.0 (± 9.2)	1239 (± 255)	9.10 (± 4.18)
≥ 20 NT, < 4 POP	7.3	2.18 (± 0.51)	38.4 (± 9.9)	1169 (± 268)	9.88 (± 4.05)
< 20 NT	53.6	2.25 (± 0.44)	39.8 (± 8.2)	1176 (± 218)	10.40 (± 4.70)
Type and quality of dental prosthesis					
FPD	17.3	2.42 (± 0.50)	41.6 (± 10.0)	1261 (± 292)	8.26 (± 4.37)
RPD					
Acceptable	31.8	2.23(± 0.47)	39.1 (± 8.8)	1178 (± 239)	9.11 (± 3.86)
Unacceptable	19.1	2.24 (± 0.41)	39.4 (± 7.9)	1181 (± 200)	10.86 (± 4.50)
CD					
Acceptable	15.4	2.29 (± 0.45)	40.8 (± 7.7)	1189 (± 215)	10.12 (± 4.53)
Unacceptable	16.4	2.33 (± 0.45)	41.0 (± 9.0)	1210 (± 239)	11.50 (± 5.16)

*Significant difference at *p* < 0.05 determined by one-way ANOVA or independent t-test

After adjusting for age and sex, the multivariable linear regression analyses revealed a significant association between dental and prosthesis status, and body composition (Table 2). There was a significant dose–response relationship between tooth loss severity and the decrease in bone mass, muscle mass and basal metabolic rate. Bone mass, muscle mass and basal metabolic rate were decreased in FPD, RPD to CD wearers, respectively. According to the adjusted beta-coefficient values and when compared to the acceptable removable denture wearers, the unacceptable denture wearers appeared to have lower muscle mass (adjusted beta-coefficient (95% CI)), unacceptable RPD = − 0.75 (− 1.14, 0.20), unacceptable CD = − 0.82 (− 0.88, − 0.76)), lower basal metabolic rate (unacceptable RPD = − 14.8 (− 38.0, − 9.21), unacceptable CD = − 11.1 (− 12.4, − 8.98)), and higher visceral fat (unacceptable RPD = 14.8 (9.21, 38.0), unacceptable CD = 11.1 (8.98, 12.4)).

**Table 2:** Associations between dental status, type and quality of dental prosthesis and body composition measurements using multiple linear regression (adjusted β (95% CI))

	Bone (mg)		Muscle mass (mg)		Basal metabolic rate (kcal)		Visceral fat (%)	
	Model 1	Model 2	Model 1	Model 2	Model 1	Model 2	Model 1	Model 2
Remaining NT and POP								
≥ 20 NT, ≥ 4 POP	0 (ref)		0 (ref)		0 (ref)		0 (ref)	
≥ 20 NT, < 4 POP	− 0.13 (− 0.34, − 0.03)*		− 1.44 (− 2.40, − 0.50)*		− 28.6 (− 50.8, − 6.5)*		− 0.23 (− 1.57, 1.11)	
< 20 NT	− 0.22 (− 0.34, − 0.10)*		− 1.67 (− 3.28, − 0.10)*		− 44.6 (− 82.1,− 7.13)*		1.18 (− 1.11, 3.47)	
Type and quality of dental prosthesis								
FPD		0 (ref)		0 (ref)		0 (ref)		0 (ref)
RPD								
Acceptable		− 0.17 (− 0.34, − 0.01)*		− 1.11 (− 2.34, 0.13)^†^		− 29.3 (− 58.3, − 0.26)*		1.04 (0.03, 2.05)*
Unacceptable		− 0.19 (− 0.38,− 0.01)*		− 1.86 (− 3.40,− 0.33)*		− 44.1 (− 80.3, − 9.47)*		1.25 (0.04, 2.40)*
CD								
Acceptable		− 0.32 (− 0.53, − 0.12)*		− 1.84 (− 3.46, − 0.22)*		− 35.9 (− 74.1, 2.42)^†^		0.46 (− 1.06, 1.58)
Unacceptable		− 0.30 (− 0.51, − 0.10)*		− 2.66 (− 4.22, − 1.10)*		− 48.3 (− 85.2, − 11.4)*		1.51 (0.23, 2.79)*

adjusted β, adjusted beta coefficient; ref, reference

*p < 0.05, ^†^0.05 < *p* < 0.10. Adjusted for age group and sex

In addition, body composition variables, peanut particle size measurements and OIDP scores were significantly increased with an augmented severity of tooth loss and growing acceptability rate from FPD to CD group (Table 3). According to the adjusted beta-coefficient and incidence rate ratio values, the unacceptable removable denture wearers exhibited a larger peanut particle size and higher OIDP score, compared with the acceptable denture wearers. According to the Spearman’s correlation coefficient (rho), bone mass, muscle mass, basal metabolic rate and visceral fat were highly correlated, but neither of them was related with OIDP scores nor peanut particle size measurements (Table 4).

**Table 3:** Associations of dental status, type and quality of dental prosthesis with peanut particle size measurement and OIDP score using multiple linear regression (adjusted β (95% CI)) and negative binomial regression (aIRR (95% CI))

	Peanut particle size (mm); adjusted β (95% CI)		OIDP score; aIRR (95% CI)	
	Model 1	Model 2	Model 1	Model 2
Dental status				
≥ 20 NT, ≥ 4 POP	0 (ref)		1 (ref)	
≥ 20 NT, < 4 POP	0.28 (− 0.39, 0.94)		1.41 (0.26, 7.70)	
< 20 NT	0.74 (0.35, 1.11)*		3.84 (1.45, 10.1)*	
Type and quality of dental prosthesis*				
FPD		0 (Ref)		1 (ref)
RPD				
Acceptable		0.25 (− 0.22, 0.72)		1.06 (0.30, 3.71)
Unacceptable		0.50 (− 0.07, 1.06)		8.98 (3.67, 40.2)*
CD				
Acceptable		0.86 (0.28, 1.44)*		7.06 (3.21, 31.1)*
Unacceptable		1.77 (1.18, 2.37)**		17.9 (6.52, 60.5)**

adjusted β, adjusted beta coefficient; aIRR, adjusted incidence rate ratio; ref, reference

*Significant association at *p* < 0.05. Adjusted for age group and sex

**Table 4:** Associations between body composition, peanut particle size measurements, and OIDP score using Spearman’s correlation (rho)

	Bone (kg)	Muscle mass (kg)	Metabolic rate (kcal)	Visceral fat (%)	Peanut particle size (mm)
Peanut particle size	− 0.05	− 0.03	− 0.07	0.16	
OIDP score	− 0.02	− 0.003	− 0.05	0.08	0.44*

*Significant correlation at *p* < 0.05

## Discussion

To our knowledge, this was the first study to determine the impact of dental status, and types and quality of dental prostheses on body composition measurements. Such body measurements are relevant biomarkers and indicators of an individual’s general health [35]. Our findings indicate that dental and prosthesis status were associated with body composition, masticatory performance and OHRQoL. Participants wearing an unacceptable RPD had a poorer OHRQoL compared with those with an acceptable CD. Moreover, masticatory performance and OHRQoL were moderately associated. However, their associations with body composition could not be found.

Further, study participants with fewer NTs and POPs tend to possess lower bone mass measurements. Regarding the ones with dental prosthesis, wearing removable dentures resulted in lower bone mass comparing with those with FPD. This might be in part due to preexisting mandibular bone loss. A previous report supported this paradigm that complete edentulism can lead to greater mandibular bone loss than partial edentulism [36]. However, previous reports targeting a female sample population found no association between tooth loss and bone mass density [19, 20]. This is perhaps due to bone mass biological variations in females throughout aging which can be affected by other relevant factors such as hormonal (through menopause, perimenopause and postmenopausal stages) and oral hygiene factors [19, 20, 37]. In the literature, it has been reported that individuals with a lower number of natural teeth or those wearing removable dentures can present a lower bone mass, which can unfortunately lead to the development of osteopenia or osteoporosis [15].

Fewer NTs and POPs were associated with lower muscle mass and higher visceral fat. A previous study in older adults also found a positive correlation between muscle mass and posterior occluding teeth determined by the Eichner’s index [21]. Even though there has been no study determining the direct association between tooth loss and visceral fat, a systematic review has shown that tooth loss is associated with obesity [38]. In addition to tooth loss, our study found that removable denture wearers showed a lower muscle mass and higher visceral fat when comparing with the ones with FPD. This finding was even more prominent if their dentures had an unacceptable retention or stability. Thus, tooth loss and unacceptable denture quality should be of concern because patients with severe muscle mass reduction may develop a sarcopenic condition [5]. Meanwhile, an increased visceral fat could lead to obesity, a risk factor for metabolic syndrome or cardiovascular disease [6].

Similar with muscle mass and visceral fat, lower basal metabolic rate was associated with less natural teeth, posterior occluding pairs, as well as type and quality of dental prosthesis. Basal metabolic rate is associated with lean body mass which refers to all body components except fat [39]. However, it can be also affected by intrinsic individual factors such as levels of physical activity [40] and circulating hormones [39]. Future studies should include these confounders to confirm the association between basal metabolic rate and oral and dental prosthesis status.

As supported by previous studies [3, 41] individuals with more natural teeth and posterior occluding pairs exhibit better masticatory performance and OHRQoL. Also, an enhanced masticatory performance and OHRQoL was seen mainly in patients with FPD, followed by RPD and CD ones. Moreover, this study found that removable dentures with unacceptable retention or stability can negatively impact OHRQoL on a more striking way than those with acceptable dentures.

The temporal association of dental status, types of dental prosthesis, and removable dental prosthesis quality with alteration in body composition could be explained by some biological plausibility. Tooth loss and ill-fitting dentures could result in declined masticatory efficiency and dietary problems such as low diet quality and nutrients intake [10, 18, 21]. Thus, changes in body composition may occur. Alteration in body composition beyond their normal limit could lead to comorbidities and affect a person’s quality of life [5, 6, 15].

In this study, body composition was neither associated with masticatory performance nor OHRQoL. However, some studies found that masticatory impairment was associated with the declined bone mass [42], muscle mass [22], and increased body fat [13] which can lead to an impaired health-related quality of life [16–18]. It might be explained by the fact that those study participants were patients who have already developed comorbidities such as osteoporosis, sarcopenia, and obesity prior to receiving dental treatment. On the other hand, the participants in our study were mostly independent, or had none or only mild systemic disease.

There are certain limitations that should be considered in this study. Since this was a cross-sectional study design, and the body composition was measured only after prosthodontic treatment, the relationships between body composition and dental status as well as prosthesis quality were limited to temporal associations but not a causal-effect relationship. Also, there is no current standard cut-off value for body composition in Asian populations. Thus, sensitivity analysis cannot be performed to determine impaired masticatory function and quality of life from body composition. Future longitudinal cohort studies are necessary in Asian populations to determine cut-off points in body composition standard measurements (e.g. bone mass) that would be most appropriate to predict masticatory function, new comorbidities and the influence of preexisting comorbidities as confounders.

Specific clinical and oral health policy implications can be suggested from our study findings. Since tooth loss can negatively affect general health of an individual, minimal intervention dentistry should be implemented to preserve natural dentition [43]. Since denture quality, particularly retention and stability, can affect both oral and general health [10, 44], healthcare providers should also focus on dental prostheses periodic follow-ups. Herein, oral and dental prosthesis status were associated with general health standard parameter, and this highlight the importance of a multi-disciplinary approach to maintain a healthy longevity and quality of life with the aging phenomenon.

## Conclusions

In individuals wearing dental prosthesis, severity of tooth loss, and types and quality of dental prostheses (retention and stability factors) could negatively impact not only masticatory function and OHRQoL, but also their overall body composition compromising their general health.

## Supplementary Information

**Additional file 1**. Raw data of the present study

## Data Availability

Dataset generated during the current study is available in the supplementary file (Additional file 1).
